# Selecting Molecular Recognition. What Can Existing Aptamers Tell Us about Their Inherent Recognition Capabilities and Modes of Interaction?

**DOI:** 10.3390/ph5050493

**Published:** 2012-05-18

**Authors:** Qian Zhang, Ralf Landgraf

**Affiliations:** Department of Biochemistry and Molecular Biology, Sylvester Comprehensive Cancer Center, Miller School of Medicine, University of Miami, Miami, FL 33101, USA

**Keywords:** aptamers, molecular recognition, nucleic acids

## Abstract

The use of nucleic acid derived aptamers has rapidly expanded since the introduction of SELEX in 1990. Nucleic acid aptamers have demonstrated their ability to target a broad range of molecules in ways that rival antibodies, but advances have been very uneven for different biochemical classes of targets, and clinical applications have been slow to emerge. What sets different aptamers apart from each other and from rivaling molecular recognition platforms, specifically proteins? What advantages do aptamers as a reagent class offer, and how do the chemical properties and selection procedures of aptamers influence their function? Do the building blocks of nucleic acid aptamers dictate inherent limitations in the nature of molecular targets, and do existing aptamers give us insight in how these challenges might be overcome? This review is written as an introduction for potential endusers of aptamer technology who are evaluating the advantages of aptamers as a versatile, affordable, yet highly expandable platform to target a broad range of biological processes or interactions.

## 1. Introduction

Aptamers, small peptides or nucleic acid sequences with unique and usually selected functional properties, have rapidly taken an important place in the available repertoire of pharmacological or diagnostic reagents. The term “aptamer” (literally meaning particles that develop a fit, “aptus”) may in its general sense apply to peptides, nucleic acids or chimera. However, it is most commonly associated with nucleic acid aptamers which are uniquely suited for SELEX (Systematic Evolution of Ligands by Exponential Enrichment). Initially employed for the study of regulatory mRNA in phage [1] and the inherent ability of RNA to form small molecule binding pockets [2], SELEX is still the most widespread method of aptamer creation (see [3] for a review of the evolution of SELEX technology since 1990). The central aspect of classic SELEX is the enrichment of desired aptamers by the partitioning method of choice followed by enzymatic reamplification. Selection criteria can be very broad from basic *in vitro* or cell surface binding to enzymatic activity, cellular uptake or aptamer survival. Aptamers can rival antibodies in affinity and specificity, providing low nanomolar or even picomolar affinity, yet they are also uniquely distinct from proteins in their mode of binding. New applications of aptamers are literally emerging every day, as are improved methods of selection. The current review will instead introduce, or reintroduce, some basic questions with respect to the fundamental molecular recognition properties of aptamers and their relation to the basic building blocks used for aptamer creation. This overview can by its very nature not be exhaustive. While some areas, such as aptamer based *in vitro* diagnostics or technical aspects of selection and minimization, are covered in great abundance in the literature, it can be much harder for a putative enduser to find at least an entry level discussion of other questions. Can aptamers target carbohydrate targets with antibody-like affinity? Is it possible to target a basic protein or peptide and still retain specificity? Can targets with negative net charge be effectively selected against while retaining specificity? The purpose of this review is to raise some of these questions and to initiate a discussion of these questions for potential users. In raising these questions, we are particularly mindful of the large divide that often seems to exist between the perceptions about aptamers in the scientific community at large and the views among established aptamer users. We also regard the relative simplicity of conventional SELEX as one of its major advantages that opens up aptamer selection to a broad audience of users. Our discussion is therefore geared more towards classic SELEX methodology that most laboratories with a primary focus on questions of biochemistry, molecular recognition or cell biology would be able to implement in a relatively cost effective manner.

## 2. General Properties of Aptamers as a Reagent Class—Why Choose Aptamers as a Design Platform?

Once non-essential sequence components are removed, aptamers tend to be approximately 1/10 the size of the average IgG antibody, another rapidly expanding class of therapeutics. This size of 7–15 kD and the phosphodiester backbone places aptamers in many ways in a niche between small molecule drugs and antibody therapeutics. Unlike most small molecule drugs, aptamers cannot readily penetrate cells unless equipped with specific uptake enhancing features that may facilitate cellular uptake. Compared to antibodies, their smaller size gives aptamers enhanced tissue penetration properties but also faster renal clearance. Modifications such as cholesterol or PEG attachment are therefore often applied to enhance serum half-life [4]. For aptamers designed to be added exogenously into a complex biological setting, increased nuclease resistance is critical. This is mostly achieved by blocking the termini of aptamers against attack by exonucleases and by enhancing resistance to endonucleases through the use of modified backbone moieties. The technical details of these frequently used modes of stabilization are not the topic of this review. However, they have implications for aptamer functions and molecular recognition as discussed briefly below.

Arguably some of the strongest aspects of aptamers are the relative ease and low cost of classic SELEX, the ease of transition from selection to synthetic production, and the versatile repertoire of defined chemical modifications for the synthetic end product. These aspects have been the driving forces for the extremely innovative research in a large number of relatively small research labs. While this has resulted in a broad spectrum of analytical applications or early studies of therapeutic usages, it stands in contrast to the relatively modest number of established clinical applications. The therapeutic application of aptamers has in part been held back by the challenges involved in the cost effective upscaling of aptamer synthesis from the laboratory scale to one that meets the reagent requirements of clinical trials. Few roadmaps existed for the large scale GMP grade synthesis and purification of nucleic acid based biopolymers. New chemical modifications continue to improve basic features of aptamers, but each new modification creates additional questions that have to be addressed in preparation for preclinical and clinical tests. As a consequence, existing therapeutic aptamers do not reflect the diversity of chemical platforms that is represented in *in vitro* applications. This complexity stands in contrast to antibodies. For therapeutic antibodies, reagent class specific questions on safety, purity, or production have been addressed. This allows evaluations to focus more rapidly on target specific studies. However, with the introduction of improved coupling methods and decreasing production costs for the most common forms of aptamers [5], we are likely to see the long anticipated increase in clinical aptamer utilization in the near future.

At present, neither peptide based approaches nor aptamers can compete with most small molecule drugs when it comes to cellular uptake. Some success has been obtained through the fusion of entry mediating peptides [6] or the targeting of internalizing surface markers ([7] and reviewed in [8]). However, with the notable exception of lysosomal enzyme replacement therapy [9], uptake and especially endosome escape remain a challenge for intracellular targets. The direct enrichment of cellular uptake as a distinct selection criterion demonstrates possible routes to aptamers that can overcome these challenges [7,10,11]. This is often combined with established approaches aimed at enhancing endosomal escape [12,13]. Since an important feature of aptamers is their modular nature, improvements in uptake methodology can in many cases be retroactively added to aptamers with previously confirmed functions. As a consequence, most proof of principle studies in complex systems involve the adaptation of previously described aptamers to novel delivery platforms. While this may seem obvious to experienced aptamer users, this relative ease of combining functionalities does stand in sharp contrast to the creation of functional fusion proteins that is often plagued by more tedious optimizations in aspects such as recombinant fusion production, folding, or linker optimization. Chimeric constructs of recombinant proteins and non-protein based moieties may be obtainable on a laboratory scale, but are challenging to produce in a defined manner at a larger scale.

Chimeric aptamers that combine distinct selected functions may initially encounter ambiguous folding as well. However, if the problematic sequence components cannot be readily identified the required optimization of fused properties can be addressed directly by SELEX itself. This concept was demonstrated early by Burke and Willis who optimized fused aptamers against very divergent cytoplasmic small molecule targets [14]. Another critical question is how much cargo needs to be delivered for efficacy if the delivered nucleic acid encoded functionality is catalytic of otherwise amplified in nature. As one example, the delivery of RNAi sequences through bifunctional aptamers has emerged as a powerful and very versatile route to future therapeutic approaches [15,16,17,18]. While the discussion of this technology is beyond the scope of this review, it is important to note that apart from the delivery efficiency of the targeting aptamer, recent studies emphasize instead the efficiency of RNAi processing, its sensitivity to the sequence context, and the use of modified bases as limiting steps.

The analysis of aptamer properties in biological systems is in most cases a more time consuming and costly undertaking than the initial selection. The ability to retroactively enhance the functionality of aptamers already under investigation is therefore of considerable advantage. In contrast to small molecule drug optimization, ongoing improvements in stabilization, pharmacokinetics or label attachment are largely substance class specific and will not have to be investigated exhaustively by each end user of aptamer SELEX methodology. Aptamers are generally considered non-immunogenic. Despite the ability of short stretches of double stranded RNA to activate Toll-like receptors, aptamers are poor activators of an innate immune response, and this response is further reduced by chemically modified RNAs [19,20,21,22]. Hence, no “humanization” of aptamers is required in contrast to antibodies. Lastly, compared to antibodies or indeed most protein display technologies, the final size of aptamers is usually well within range of commercially accessible chemical synthesis. The synthesis and validation of aptamers that carry an exactly defined number of fluorescent probes, crosslinkers or prodrugs at clearly defined positions is relatively simple while this can be a very labor intensive task for other carriers such as antibodies. This relative ease by which functional groups can be introduced into aptamers is not only of importance for their use as *in vitro* diagnostics, but greatly enhances the utility and evaluation of therapeutic aptamers through the introduction of fluorescent probes in mechanistic cell culture studies or the introduction of tracers in pharmacokinetic and imaging applications.

## 3. The Choice of Building Blocks

A survey of aptamer selections over the years reveals a strong initial bias towards ribonucleotides as building blocks despite the inherent enzymatic and chemical instability of unmodified RNA. This initial preference for RNA over DNA largely reflects the believe that the expanded ability of RNA to form complex three dimensional structures is more likely to mimic the structural repertoire of proteins. Due to the subsequent introduction of modified and more nuclease RNA, current RNA derived aptamers exceed DNA in serum stability. However, DNA aptamers have become more popular in recent years, largely due to a selection procedure that does not require an intermediate reverse transcription step, the relatively high intrinsic serum stability of DNA without the need for modified deoxyribonucleotides, and the low cost and high yield in the synthetic production of final products. In addition, the documented ability of select DNA aptamers to rival their RNA counterparts in affinity demonstrates that the lower structural complexity of DNA may be much less of an inherent limitation than was initially believed. At present, existing DNA aptamers exhibit a strong bias towards quadruplex structures or three-way junctions. Whether the apparently more restricted structural space of DNA aptamers will ultimately translate into a more limited molecular recognition space remains to be seen. So far, this has not emerged as an obvious limitation.

**Figure 1:**
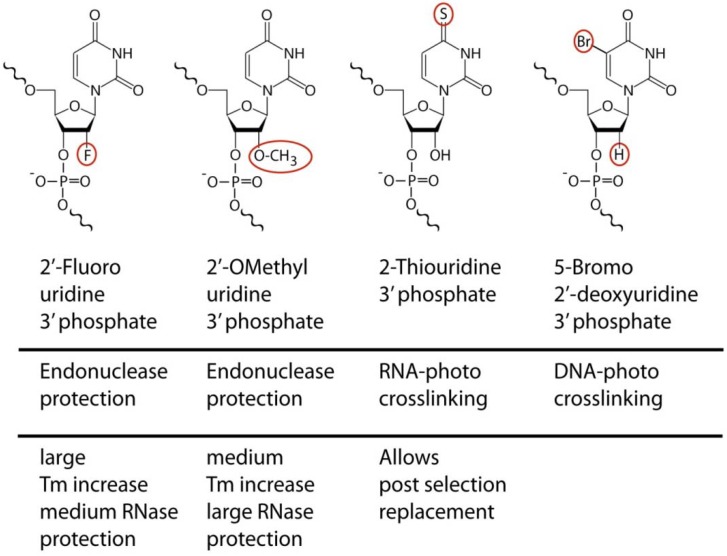
Examples of chemical modifications that provide additional functionality to aptamers and that are with varying efficiencies compatible with enzymatic incorporation. The aptamer related usage and noteworthy features relevant to molecular recognition are indicated.

For RNA aptamers, considerable efforts have been placed into methods of stabilization. While various capping schemes protect aptamers from enzymatic endonuclease attacks, and a broad range of chemical modifications are available for synthetic RNA oligonucleotides, the dominant SELEX compatible modifications use 2'-fluoro or 2'-*O*-methyl modified ribonucleotides (Figure 1). The choice of platform has significant functional implications beyond stability considerations. The retroactive replacement of nucleotides for modified derivatives significantly alters aptamer structure and function. Traditional methods involving the iterative cycles of replacement and functional analysis have given way to selection procedures that incorporate modified bases directly during the SELEX procedure, yielding aptamers that are effectively unrelated to their unmodified counterparts in binding properties. Replacement of pyrimidines alone achieves already significant stabilization, although this is dependent on the spectrum of nucleases being tested.

A subset of nucleases with exceptionally high ability to digest even modified RNAs is found in various strains of mycoplasm unless 2'-*O*-methyl substitution is applied not only to pyrimidines but all nucleotides [23]. In retrospect, the relative stability of *O*-Methyl modified RNA has to be viewed in the context of its natural occurrence. 2'-*O*-methyl modifications of RNA were found early (1966) in *E. coli* [24], and the specific methylation products were later identified in rat liver RNA (1972) [25]. Interestingly, the dominant substrate of enzymatic 2'-methylation in these studies of alkaline resistant RNA turned out to be purines. So far, the higher incorporation yield of mutagenized polymerase for 2'-fluoro modified NTPs has been the main reason for the relative dominance of 2'-fluoro over 2'-*O*-methyl modification to achieve serum stabilization. The drawback of this approach is the significantly higher cost and technical challenge involved in the chemical synthesis of the 2'-fluoro-substituted RNA end product. The latter is no significant impediment for 2'-*O*-methyl modified RNA. T7 polymerase mutants have been described that produce 2'-*O*-methyl RNA [26] as has the direct selection of 2'-*O*-methyl modified aptamer against VEGF [27]. However, the high optimization requirement for the 2'-*O*-Me accepting polymerase reaction has until recently remained a limiting aspect to its widespread use.

RNA or DNA Spiegelmers are an alternative solution to the problem of aptamer stabilization, conceptually akin to anti-idiotype antibodies. Spiegelmers (mirror images) are the synthetic enantiomers (e.g., L-RNA) of conventional aptamers that were initially selected against the mirror image of the final target. Spiegelmers against L-arginine were an early examples [28]. Since most enzymes, including RNases, evolved to act on the natural D-enantiomers of nucleic acids, Spiegelmers exhibit excellent serum stability (reviewed in [29]). While Spiegelmers and their serum stability have been tested for peptide size targets such as gonadotropin-releasing hormone (GnRH) [30,31], the broader utilization of this technology has so far been limited by the requirement to synthesize the target of interest in its enantiomeric form. The subsequent selection follows a standard SELEX approach. The ability to dissect out a smaller segment of the target protein for the initial selection has been demonstrated against the 28 kD staphylococcal enterotoxin B [32]. However, while conceptually intriguing, Spiegelmer creation remains relatively labor intensive by comparison to direct methods of serum stabilization.

In addition to targeting well established biomarkers such as PSMA for cargo delivery [18,33], aptamers have proven powerful tools in the *de novo* identification of biomarkers on live cells [7,34,35], or in the dissection of the cell surface context of signaling events [36]. The latter is aided by the ability to enzymatically incorporate photo crosslinking moieties such as 5-iodouracil [37], BrdU [19] or thio-Uracil. In contrast to the unrelated 2'-ribose modifications, the 2-thiouracil base modification is unique in that it represents a naturally existing base substitution for RNA. The replacement of uracil by thiouracil (Figure 1) does not change the affinity or specificity of the aptamer and hence provides a rare example of a functionally neutral post selection replacement. This allows very selective cross linking procedures at high UV wavelength (330 nm) using aptamers that were initially selected without photo reactive groups [36]. These and other modification options for aptamers facilitate access to many early proof of principle studies before obstacles of converting aptamers into *in vivo* optimized reagents need to be tackled.

## 4. Beyond Nuclease Resistance—Secondary Consequences of Stabilizing Substitutions

**Figure 2:**
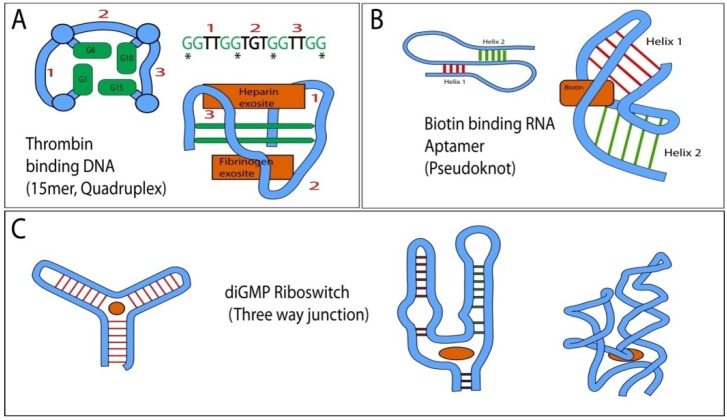
Topologies frequently found in RNA and DNA aptamers and their simplified 3D structures with indicated location of ligand binding sites (orange). (**A**) 15 nucleotide core of the Thrombin binding quadruplex DNA aptamer [42,43,44]. The central structural feature are two layers of quadruplex guanosines, derived from the repeating GG motif of the primary sequence (interacting guanosines in left representation are marked by “*” in the primary sequence). The aptamer can interact with either the fibrinogen or heparin exosite and crystallized “sandwiched” between two thrombin molecules. The connectivity of the intervening loops (1–3) differs between quadruplexes and loop sequences provide binding specificity; (**B**) Biotin binding pseudoknot RNA aptamer (adapted from [45]). The interface of the two tilted helices provides a cavity in which biotin binds; (**C**) Left: Generalized topology of a three way junction and identification of its central ligand binding cavity. Right: Simplified 2D and 3D structure of the diGMP binding Riboswitch (adapted from [46]). Note how in this example, the addition of unpaired nucleotides increases both the size and the interaction potential of the central cavity in the three way junction.

Whether or not functionality in a cellular setting is ultimately desired has important consequences in the choice of the selection platform. The introduction of 2'-ribose modifications does alter folding, such that aptamers selected with standard nucleotides cannot be readily translated into their corresponding counterparts post selection, or *vice versa*. In addition, the biophysical properties of the enzymatically incorporated 2'-fluoro and 2'-*O*-methyl modified aptamers are fundamentally different on various functionally relevant levels, maybe most importantly their hybridization energy. Average melting temperatures of homo duplexes increase in the order DNA < RNA < 2'-*O*-methyl-RNA < 2' F-RNA [38,39]. This alters the intrinsic conformational flexibility of modified aptamers in ways that may influence the range of capabilities. While RNA is capable of catalytic functions and can bind its respective substrates with high affinity, product release has long emerged as the dominant rate limiting factor for catalytic RNA enzymes. One example is provided by drug sensing hammer head ribozymes for which the rate of allosteric transition has been shown to be rate limiting [40]. On the other hand, loss of stability due to insufficient magnesium complexation results in inactive and excessively flexible vEGF aptamer [41]. The polymerase tolerated ribose modifications make existing aptamers more rigid while more bulky base modifications are expected to have the opposite effect. The importance of controlled flexibility, well established as a key contributor to the ability of proteins to recognize and bind their targets, is not surprising given the broad range of complex 3D structures that have emerged for various aptamers or ribozymes (examples in Figure 2).

Hence, additional means of fine tuning hybridization could give aptamers a broader range of conformational dynamics, much as proteins can evolve to accommodate a broad range of demands on their intrinsic flexibility depending on their operating environment. However, the functional impact of these modifications on aptamer functions other than nuclease resistance is at present rarely exploited in a systematic manner, largely due the difficulties involved in optimizing the enzymatic incorporation of modified building blocks during classic, amplification dependent SELEX.

## 5. Molecular Recognition—Are All Targets Equally Accessible?

The challenges aptamers face in molecular recognition are obviously heavily dependent on the reagent class to be targeted. Many applications of aptamers make use of their intrinsic ability to base pair to nucleic acid targets. The rules governing specificity in these cases are relatively well understood. For the previously mentioned use of aptamers as tools in siRNA delivery, sequence components providing siRNA functionality must strike a balance between stability and processing by the cellular machinery. However, while hybridization driven functionality is a very prominent feature for aptamers, especially for aptamers used for *in vitro* diagnostics, most aptamers used in a biological setting represent or incorporate targeting modalities aimed at non-nucleic acid ligands. Small molecule binding aptamers and the control of mRNA processing are combined when the aptamer is introduced into the UTR region of mRNA. In such chimeric constructs, the aptamer can directly regulate translation [47] or can control splicing in the case of allosteric riboswitches (early design in [48], reviewed in [49]). More recent examples of riboswitches include the theophyllin [50] or tetracycline [51] induced splicing of aptamer-mRNA hybrids, a concept directly taken from naturally existing riboswitches in bacteria, fungi and plants were they regulate protein expression or splicing in response to small molecule stimuli. In these and other settings that do not use conventional hybridization between the aptamer and the “ligand”, aptamers act effectively like ligand (or epitope) binding proteins. Given that proteins and nucleic acid aptamers differ fundamentally in the inherent chemical complexity of their building blocks this has long prompted the question whether both employ similar modes of target recognition or whether specific classes of targets may be less suitable for aptamers. While a systematic comparative experimental analysis of this question is at present not available, some insight can be obtained from the range of available aptamers, especially in cases were structural data are available to dissect the mode of recognition. Small molecule recognition was the focus of many initial aptamer selections and provided a wealth of highly selective, high affinity aptamers (reviewed early on in [52]). One of the earliest examples highlighting the achievable affinity and selectivity of aptamers were aptamers against theophyllin. Final RNA aptamers did not only bind with K_d_s of 100 nM and 600 nM but discriminated against the closely related caffeine 10^4^-fold. More importantly, this represented a more than a 10-fold improvement in discriminating power compared to antibodies [53]. While the repertoire of aptamer targeted compounds is very large by now, some ligand classes, such as organic dyes or nucleotide derivatives, are clearly more abundant than others. How do the general biochemical characteristics of the target influence the probability of successful aptamer selection?

## 6. Hydrohopic Targets

While proteins have a series of side chains at their disposal for contacts with hydrophobic targets, nucleic acids are more limited in their repertoire of available interactions. A therefore somewhat surprising example for the formation of an exclusively hydrophobic ligand binding pocket was found in an aptamer that binds the acyl component of the phosphoglycolipid antibiotic moenomycin A with high (400 nM) affinity [54]. While fully solvent exposed acyl chains are uncommon in an *in vivo* setting, aptamers have also been selected against both alkaloids and steroids. The latter are largely variations of the well studied and conformational switch capable cocaine binding DNA aptamers, an example of a three way junction. In its most basic form, three way junctions provide a hydrophobic binding pocket at the central branching point (Figure 2C, left) [55]. Here the three converging helices project their respective terminal base pairs towards a central cavity with an estimated diameter of 10–15 Å. (reviewed in [56]). With a cholic acid binding DNA aptamer as the starting point, mutagenesis revealed that effectively all changes near the junction influenced affinity but not specificity. Permutations of the six nucleotides that make up the three base pairs of the minimal central cavity allowed to discriminate cholic acid from more hydrophobic steroids such as androsterone. Final affinities were however modest and in the micromolar range [57,58]. The generic 2D representation of a three way junctions in Figure 2C highlights the location and nature of the central binding pocket, but is misleading. Hydrodynamic studies of the steroid hormone binding DNA aptamer indicate significant ligand-induced structural changes leading to a more compact fold [59]. A structurally well resolved and divergent three way junction is exemplified by the diGMP binding riboswitch (Figure 2C right). It shows an expanded central cavity due to mismatched nucleotides and bulges that provide both additional space and interaction potential. A good example of the underutilization of hydrophobic interactions compared to proteins is provided by the RNA pseudo knot structure that binds biotin with an affinity of about 6 μM [60]. This aptamer binds biotin at the interface of two helices that constitute the pseudo knot (Figure 2B) through interactions with the hydrogen base pair capable head group of biotin. The long fatty acid chain of the cofactor is not utilized [45]. This is in stark contrast to avidin or streptavidin (K_D_ approx. 10^−15^ M) which bind biotin in a buried binding site through a channel that maximizes interactions with the hydrophobic linker.

## 7. Carbohydrates

SELEX has generated various examples of selective binding to carbohydrates (reviewed in [61]). As was the case for hydrophobic groups, selectivity can be achieved, but thus far, final affinities are largely in the micromolar range. While carbohydrates provide opportunities for hydrogen bonding, affinity may be reduced due to their inherent flexibility and absence of planar, aromatic ring structures or positive charges in most cases. Not surprisingly, some of the best results have been obtained against amino glycosides such as neomycin B, which is recognized by an RNA aptamer with a 100 nM affinity [62]. However, most affinities obtained so far are rather modest with dissociation constants between 10^−6^ and 10^−4^ M. These include challenging proof of principle targets such as the selective binding of various monosacharides [63] or cellubiose [64] for which the tightest binding aptamer achieved an exceptionally low K_D_ of 10^−7^ M. While most affinities remain relatively modest compared to other aptamer targets, selectivity is in most cases very high providing good potential for catalytic aptamers or multivalent homo or bifunctional aptamers that may bind complex carbohydrate polymers with high affinity. Low micromolar binding affinities also have to be viewed in the context of relevant biological concentrations of metabolites and the affinities against single carbohydrate units generated by proteins. Glucose binding protein, first found in the periplasmic space of *Pseudomonas aeruginosa*, is considered to be a very sensitive and selective sensor of glucose in the extracellular milieu. It binds glucose with a K_D_ of 0.35 μM and withstands challenges by access competing carbohydrates in 10-fold but not 100-molar excess [65]. Given that glucose concentrations of diagnostic and functional relevance in mammals are in the milimolar range, the already existing selectivity of aptamers for specific carbohydrates may ultimately be more important than the low micromolar affinities. A particularly challenging target for aptamers is represented in Silalyllactose. This target includes a carboxyl group and provides an example for the use of modified bases to overcome the challenge of additional repulsion by negative charge. The selection of DNA aptamers with amino functionality carrying thymidine triphosphate during selection provided aptamers that achieved comparable affinity to non-charged carbohydrates (K_D_ = 4.9 μM) and provided an important proof of principle for expanded SELEX with charge modified nucleotides [66].

## 8. Proteins/Peptides

A 2'-fluoropyrimidine modified RNA aptamer (NX1838) against VEGF [67,68], introduced in 1999, is probably one of the best known and characterized examples of an aptamer that has moved forward into clinical usage for age related macular degeneration. Arguably, proteins and peptides represent by far the largest groups of aptamer targets with therapeutic and diagnostic relevance. We will focus on a few select examples that facilitate an analysis of the modes of recognition employed by aptamers.

Factors that control blood clotting are of great importance for the acute treatment of a large number of cases of thrombosis and have been a significant emphasis of aptamer research over the years, providing several in depth comparative studies. High affinity (20 nM) thrombin aptamers, reported in 1992 [69], were among the first reported aptamers and the first example of ssDNA aptamers. Often overlooked, but maybe as remarkable is the fact that a subsequent study of the antithrombotic properties of defibrotide, a porcine mucosa derived fragmented and mostly single stranded DNA preparation, identified short single stranded DNA segments through direct enrichment on immobilized thrombin. The enriched DNA segments resembled those obtained by SELEX in both sequence and binding properties [70]. While no novel aptamers were recovered by this approach, enrichment and sequence identification was achieved without repeated cycles of conventional SELEX. A recent biophysical comparison of the mode of binding by two of the most studied anti thrombin aptamers, DNA sequences of 15 and 29 nucleotides length, also proves insightful with regard to the diversity of binding modes generated by aptamers. Studies as late as 2011 showed that both aptamers invoked very different modes of binding with the 15-mer binding as a duplex through largely electrostatic interactions while the 29-mer utilized a quadruplex structure and predominantly hydrophobic interactions [71]. While anti-thrombin DNA aptamers were among the first to be discovered, RNA aptamers against factor IXa were the first to be evaluated for key pharmacokinetic properties in humans as part of their phase I trials, reported in 2006 [72]. Serum stable variants of anti-factor IXa aptamers are potent inhibitors of blood clotting and the nucleic acid nature of the aptamer allows for complementary sequences to be injected as fast acting antidotes. Regado Biosciences markets REG-1 as a combined Factor IXa antagonist aptamer (RB-006) and oligonucleotide activity control agent (RB-007). This concept maximizes many of the inherent advantages of aptamers and provides a precedent for aptamer-derived tunable neutralizing reagents against a variety of serum accessible or circulating targets *in vivo* [73].

The initial selection against thrombin capitalized on the presentation of positively charged epitopes representing the sites binding of fibrinogen and heparin respectively. Since many RNA binding proteins utilize arginine or lysine residues to interact with their respective targets, it is tempting to extrapolate from our knowledgebase of nucleic acid binding proteins to predict the mode of binding and limitations in specificity for aptamers. However, a structural analysis of arginine directed aptamers surprisingly reveals an avoidance of charge based interaction and instead shows a reliance on hydrogen bonding in a complex with a fully engulfed arginine side chain. This mode of recognition is in stark contrast to the reverse interaction exhibited by RNA binding proteins. It has been suggested that this reflects the intrinsically different selection pressure for aptamers that allows exclusive emphasis on selectivity and affinity without the more complex functional requirements imposed on proteins [74]. Regardless of the driving force for this selection, it underscores the difficulties involved in the logical prediction of anticipated aptamer properties obtained during selection. Although it is conceptually difficult to draw clear distinctions, the different modes in binding could be regarded in terms of the directionality in the recognition process. To the extent that aptamers are targeting relatively small “ligands” that can be “engulfed by the aptamer with a suitable binding pocket, modes of binding appear to emerge that do not reconcile with our conventional understanding of protein-nucleic acid interactions. The latter involve large and significantly solvent accessible interfaces in proteins. When aptamers are selected in a manner that favors this mode of recognition, interactions that emerge may resemble “conventional” nucleic acid—protein interactions more closely. One example is the structure of the NF-κB—RNA aptamer complex [75]. Two RNA aptamer molecules bind the NF-κB (p50) homodimer independently with high selectivity over p65. The mode of binding resembles that between transcription factors and their cognate DNA, including aspects like water-mediated readout. However, the RNA aptamers stabilize a clearly distinct conformation of the protein dimer. Hence this SELEX derived observation may point towards additional roles of NF-κB in the direct regulation of cellular RNA in a way that was aptly termed “molecular mimicry” [76].

**Figure 3:**
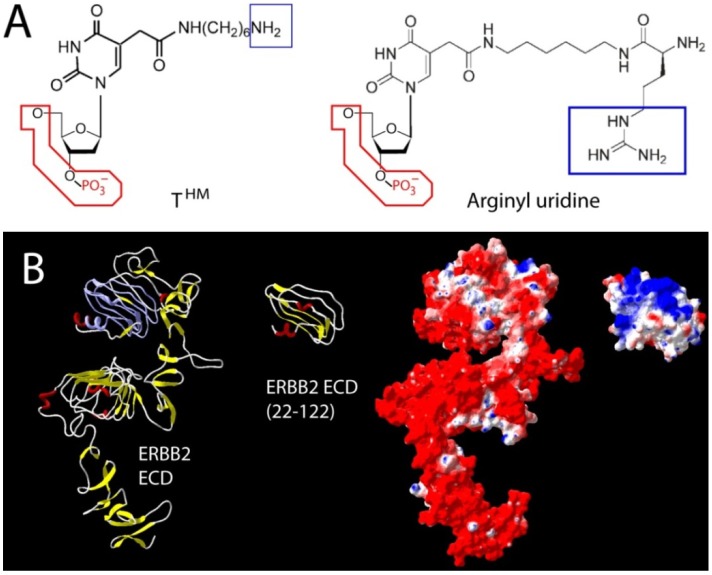
Two different approaches used to counteract charge repulsion of nucleic acid aptamers. (**A**) T^HM^ (5-*N*-(6-aminohexyl) carbamoylmethy-2-*O*-deoxyuridine) and arginyl uridine represent two modified bases that can be enzymatically incorporated. This approach yielded aptamers against Silalyllactose [66] (T^HM^), or glutamic acid [80] and carboxy glutamic acid [81] respectively (arginyl-uridine); (**B**) Aptamer selection against the extracellular domains of ERBB2 (HER2) has been challenging due to its uniformly distributed negative surface charge (red in surface representation). In the absence of its extreme *N-* and *C*-terminal capping regions, a segment (22–122) of domain one provided suitable bait for the selection of a nanomolar RNA aptamer. Once selected, the final 2' Fluoro RNA aptamer retains its high affinity *in vitro* and *in vivo* in the presence of surrounding negative surface charges [79]. Truncations and surface charge calculations of the reference structure for ERBB2 [82] were generated in Swiss PDB viewer using default parameters.

The selection of aptamers against transcription factors or other nucleic acid binding proteins can be a challenge for target regions other than the nucleic acid binding portion. Positively charged surfaces can steer aptamers towards an epitope during selection, even if the final binding mode does not directly capitalize on charge interactions. But does such a favorable attraction undermine specificity? A direct evaluation of this question in 2011 revealed that aptamers can readily discriminate a panel of basic proteins [77], thereby disproving a common perception that the presence of positive charges in a target implies an unspecific mode of binding. A more difficult challenge is presented by the inverse scenario, the selection of aptamers against targets with pronounced negative net charge in the absence of hydrophobic or aromatic interactions that could present strong compensating interactions. While an obvious impediment for small ligands, this has also been a significant limiting factor for selections against high priority therapeutic protein targets. This is exemplified in the cancer relevant ERBB receptor class. While the ERBB3 (HER3) receptor tyrosine kinase with its relatively balanced extracellular surface charge (glycosylated ECD mw = 82kD including carbohydrate) was one of the earliest large size proteins to be targeted by aptamers [78], its clinically very prominent homolog, ERBB2 (HER2) has long stubbornly resisted multiple attempts to select aptamers, in large part due to its abundant negative surface charge. Only very recently has this challenge been overcome through the use of a small defined subsegment of the receptor ECD yielding high affinity, 2'-fluoro-modified RNA aptamers (minimized synthetic aptamer K_D_ = 3.5 nM) with specificity in an *in vitro* and cellular setting [79]. While N-terminal amino acids of ERBB2 (amino acid 22–122) provided a suitable fragment for the selection, such an approach requires upfront structural insight unless costly and time consuming random dissection and validation approaches are used in conjunction with SELEX. Ideally, the target segment should be solvent accessible, sufficiently unique in composition, non-glycosylated (due to limitations in carbohydrate binding and large variations in glycosylation patterns across tissues), balanced in charge, and capable of exhibiting a biologically relevant 3D structure (or natural flexibility) when taken out of context. The example of ERBB2 selection also demonstrates that aptamers against epitopes in a highly negatively charged surrounding can be successfully created, provided the initial hurdle of charge based repulsion can be overcome during early selection cycles (Figure 3).

While this represents one viable approach to overcome the challenge imposed by very pronounced negative surface charges, an alternative and potentially more universal approach was outlined in principle by the earlier mentioned selection of modified aptamers against Sialyllactose. A similar selection using arginyl dUTP during SELEX yielded high affinity DNA aptamers against glutamic acid [80] as well as gamma carboxy glutamic acid, a calcium chelator on many blood clotting factors [81]. However, while the arginyl modifications were essential for binding and several of the aptamers displayed enantioselectivity, the final affinities were nevertheless modest. Whether a similar approach against a less challenging target with more variable recognition motifs will provide a universal route for high affinity aptamers against predominantly negatively charged proteins remains to be seen. In the meantime, structural dissection may be the method of choice to overcome the negative impact of charge repulsion in the early cycles of selection.

## 9. Aptamer GFP

A particularly interesting challenge of molecular recognition by aptamers and their ability to rival proteins in functionality involves the quest for nucleic acid GFP equivalents. A unique feature of fluorescent proteins is the spontaneous oxidation and cyclization chemistry of several core amino acids. In the case of GFP the final fluorophore resulting from this intramolecular reaction is 4-hydroxybenzlidene imidazolinone, shielded in the core of the GFP beta barrel. The limited chemical diversity of aptamers restricts options for similar chemistry, but fluorophores and other modifiers are readily attached to synthetic aptamers carrying amino or thiol functionalities. The enzymatic site directed incorporation of intrinsically fluorescent nucleotide analogs has been achieved using T7 polymerase and DNA templates in which modified bases flank the site of insertion [83]. While these and similar approaches are of special importance for mechanistic studies, it has limited applicability to live cell studies, the ultimate goal for “aptamer-GFPs”. Various attempts have been made to utilize fluorescent dyes, such as Hoechst dye derivatives [84] or Malachite green [85,86] that achieve or significantly increase fluorescence upon aptamer binding. The challenge in these approaches is the need for cell permeable cofactors that avoid intercalation based binding modes that are highly favorable for aptamers but would for the same reason result in high background binding and fluorescence to the vast excess of cellular RNA and DNA. In effect, the objective has to be the selection of a strongly fluorescence enhancing mode of binding that avoids the most obvious modes of interaction. This makes the recent report of a GFP-aptamer, termed “spinach”, particularly remarkable [87]. “Spinach” is reconstituted through the association of an aptamer core (RNA 13-2) and a cell permeable and non-cytotoxic dye [3,5-dimethoxy-4-hydroxybenzylidene imidazolinone (DMHBI)]. DMHBI achieves strong green fluorescence when bound to aptamer while showing very low cellular fluorescence in the absence of aptamer. While further improvement of color diversity, quantum yield and key benchmarks are likely to follow, the ramifications of this advance are clear in light of the ways in which few studies in the protein field are nowadays conceivable without the involvement of some form of fluorescent protein.

## 10. Aspects of Aptamers Dictated by the Selection Methodology

The requirement for enzymatic amplification in all classic SELEX approaches does by definition dictate the nature of building blocks, and indeed many aspects of existing aptamers. While randomized windows of about 40 nucleotides are still common, a complete permutation of 4^40^ or 10^24^ possible sequences is already beyond the maximum experimentally achievable complexity, which is more often around 10^13^ molecules per screen. Some recent protocols operate with randomized sequences of around 20 nucleotides and thus can at least in principle be fully represented in the experimentally accessible population of sequences carrying 4^20^ or 10^12^ permutations. In addition, the need for reamplification adds non randomized flanks that can serve as both primer sites for amplification and unique tags that can be highly beneficial if multiple SELEX procedures are being carried out in parallel. Thus, initial aptamers derived from SELEX range frequently from 50–80 nucleotides. An important first step in further analysis is the identification of the minimum size needed for function. Since the non-randomized flanking reagents can take part in the overall base pairing, it is not necessarily clear whether the randomized core does indeed represent the functional unit, and a variety of tools have emerged over the years to stream line this process of minimization (classic approaches reviewed in [88]).

While classic approaches to aptamer minimization relied mainly on the reiterative use of biochemical methods and algorithms that evaluate the energetics of alternative two dimensional folding options, more advanced approaches include direct predictions of three dimensional folds [89,90,91]. However, while the compact nature of experimentally determined 3D structures (examples provided in Figure 2) often stands in stark contrast to the 2D representations derived through simple hybridization and energy minimization schemes, the latter often provide good approximations of the underlying hybridization backbone and are readily available to most endusers. Some more contemporary approaches incorporate methods such as “primer less” amplification or the combination of high throughput sequencing and bioinformatics processing [92,93,94].

Beyond minimization approaches, one of the more profound shifts in the day to day business of aptamer selection has serendipitously arisen from the rapidly dropping costs of high throughput sequencing. This makes it for the first time possible to follow aptamer “enrichment” throughout the selection cycle on a sequence level. Data derived from such efforts are readily processed by an array of parsimony based methods, initially designed to deconstruct protein and gene evolution. As more studies are emerging, it becomes clear that many previously held believes about the underlying driving forces of selection and readouts of selection progress may have to be revisited. In addition, such studies have demonstrated the contribution of the amplification methodology to drifts in the aptamer population (neutral SELEX). Such drifts occurs in the complete absence of biochemical enrichment or partitioning steps [95]. One example is an aptamer length dependent bias against the incorporation of adenine [96].

The ability to follow the early rounds of selection directly for the emergence of sequence clusters instead of driving selection towards complete convergence has also coincided with technical advances in enrichment and detection methodology that bypass reamplification steps. While in principle already represented in the before mentioned direct selection of anti-thrombin DNA aptamers from defibrotide in 1994, a systematic exploitation of consecutive rounds of enrichment without reamplification was formally introduced by Berezovski *et al*. [97]. This “non-SELEX” approach achieved the amplification-free enrichment of DNA aptamers against H-Ras by four orders of magnitude in three enrichment steps. The limit of amplification free selection is currently determined by the mode of detection. At the same time, microfluidics has reduced sample size and processing time [98] while on chip processing has effectively set the stage for fully automated SELEX [99] or non-SELEX.

## 11. Conclusions

What does the future hold for aptamers? Traditionally, the role of nucleic acids in therapeutics has been relegated to the coding of delivered genes, possibly supplemented by the enhanced delivery of fused aptamers. An area of rapidly increasing importance is the delivery of nucleic acids as regulatory entities. In the near future, aptamers are likely to capitalize on the advances made in stabilization and targeting to deliver highly potent regulatory cargo with functions such as siRNAs in ways that are unique to nucleic acid carriers. In the more distant future, aptamers with improved catalytic functions may provide new paradigms for enzyme replacement therapy by not only enhancing delivery but by directly representing the catalytic entity itself. The question whether nucleic acids ultimately remain dominant as the basic building block in aptamer creation is directly linked to advances in enrichment, detection, characterization, and cost effective multiplexing in non-SELEX approaches. In the meantime, the classic SELEX procedure has undergone major improvements over the years but it continues to remain readily accessible to most research labs. In addition, nucleic acid aptamers are likely to profit, much like the field of antibody therapeutics did, from the fact that many critical issues, such as the fine tuning of serum stability, pharmacokinetics, or the evaluation of chemical modifications, can be evaluated for aptamers as a compound class. Hence, the current rapid growth in the use of nucleic acid aptamers is unlikely to slow down in the near future.
